# Predictors of psychiatric rehospitalization among elderly patients

**DOI:** 10.12688/f1000research.7135.1

**Published:** 2015-09-30

**Authors:** Chun Yin Terry Wong

**Affiliations:** 1 Department of Psychiatry, Pamela Youde Nethersole Eastern Hospital, Hong Kong, Hong Kong

**Keywords:** risk factors, psychiatric, readmission, rehospitalization, elderly, geriatric

## Abstract

The population of Hong Kong and the proportion of elderly people have been increasing rapidly. The aim of this retrospective cohort study is to determine predictive factors for psychiatric rehospitalization within 2 years among elderly patients who were discharged from psychiatric wards, in attempt to reduce their rehospitalization rate and to reintegrate them into the community. Patients aged 65 and over, who were discharged from psychiatric wards of Pamela Youde Nethersole Eastern Hospital from 1 March 2010 to 29 February 2012, were identified. Rehospitalization within 2 years after discharge was the primary outcome measure, and the time to rehospitalization was measured as the secondary outcome. Patients were subgrouped into readmitted and non-readmitted groups. Logistic regression and Cox regression analyses were applied to the potential predictive factors with odds ratios and hazard ratios obtained, respectively, for the significant findings. Kaplan-Meier survival curves were plotted for graphical representation of the study results in survival analysis. 368 individuals satisfying the study criteria were identified. The same four factors were shown to be significantly associated with rehospitalization in both multiple logistic regression and Cox regression survival analysis. Referral to other psychiatric disciplines upon discharge (p< 0.001, OR=0.325, HR=0.405) was associated with a lower rehospitalization risk and correlated to a longer time to rehospitalization. History of suicidal behaviors (p< 0.001, OR=4.906, HR=3.161), history of violent behaviors (p< 0.001, OR=5.443, HR=3.935) and greater number of previous psychiatric admissions (p< 0.001, OR=1.250, HR=1.121)  were associated with a higher rehospitalization risk and predicted earlier rehospitalization. The rehospitalization rate of elderly patients was 5.2% at 1 month, 9.5% at 3 months, 15.0% at 6 months, 17.1% at 1 year, 18.8% at 1.5 year and 20.9% at 2 years.

## Background

The population in Hong Kong and the proportion of elderly people are increasing rapidly. Pamela Youde Nethersole Eastern Hospital is a regional hospital under the Hospital Authority serving the eastern district of Hong Kong Island. The characteristics of elderly patients were different from that of general adults, for example, more of them suffered from cognitive disorders, they were more often hospitalized due to medical comorbidities, and they might require placement such as old age homes after discharge. Despite many psychiatric readmission studies were already available in the literature, surprisingly very few of them were targeted to elderly patients only.

Rehospitalization had been regarded as a useful indicator to measure quality of care provided by hospitals worldwide ^1, 2^. Since there are only a small number of elderly psychiatric rehospitalization studies available, studies involving elderly patents and patients of all ages (including the elderly) were reviewed. Socio-demographic factors that were identified as significant in previous studies included: age ^3– 10^, gender ^5, 8, 11– 14^, ethnicity ^15^, marital status ^14, 16– 20^, education level ^13^, and type of residence ^5, 6, 8, 10, 15, 17, 22, 23^. Clinical factors significantly associated with rehospitalization included: length of inpatient stay ^3, 5, 9, 10, 16, 19, 24, 25^, primary psychiatric diagnosis ^4, 5, 8, 9, 11, 14, 18, 19, 21, 22, 24, 26^, presence of psychiatric comorbidities ^4, 17, 27^, presence of medical comorbidity ^21, 27^, cognitive impairment ^3, 25^, referral for aftercare services ^6, 7, 29, 30^, history of suicidal behaviors ^12^, history of violence ^11, 25, 28, 31^ and number of previous psychiatric admissions ^7, 8, 15, 20, 25, 32, 33^. Many of the articles adopted a retrospective design and involved psychiatric patients of all ages, while very few were specifically targeted elderly patients ^20, 22, 27, 33, 34^, and no such data was available for elderly psychiatric patients in Hong Kong. Therefore this study was conducted to identify risk factors in attempt to reduce rehospitalization, so mental health services could be utilized more effectively in view of the increasing needs from the aging population.

## Methods

The aim of this retrospective cohort study is to determine predictors for psychiatric rehospitalization over 2 years among elderly patients after they were discharged from psychiatric wards.

### Inclusion criteria

Patients aged 65 and over, who were discharged from the psychiatric wards of Pamela Youde Nethersole Eastern Hospital from 1 March 2010 to 29 February 2012, formed the study population. For patients having repeated discharge episodes during the study period, only the first discharge episode was included as the index episode for that patient to avoid duplication of data from the same individuals.

### Exclusion criteria

Patients who died during inpatient stay at the index episode, or had not received mental health care in any psychiatric clinics under the Hospital Authority after discharge, including those who were followed up by private psychiatrists or overseas psychiatric care systems, were excluded due to a lack of information to analyze their outcome.

### Data collection

A list of patients satisfying the study criteria was generated from the Clinical Data Analysis and Reporting System (CDARS) of the Hospital Authority’s Medical Records Office. The socio-demographic and clinical factors of each patient were determined by reviewing their medical records in the Hospital Authority’s Clinical Management System (CMS) and case notes as charted by their respective case doctors. The CMS contained information on patients who had received health services under the Hospital Authority including their demographics, inpatient discharge summaries, outpatient consultation notes, physical or psychiatric diagnoses and medications that were prescribed. Data was collected electronically and entered in Microsoft Office Excel version 2010.

### Dependent variable

Rehospitalization was defined as the primary outcome.

### Independent variables

Socio-demographic factors included age (upon discharge), gender, ethnicity, marital status, education level and type of residence (upon discharge). Clinical factors included priority follow-up (PFU) status (see Appendix for more details), length of inpatient stay, primary psychiatric diagnosis (as determined from the medical coding with ICD-9-CM in CMS by respective case doctors), presence of psychiatric comorbidities (as reflected by more than one psychiatric diagnosis recorded in CMS in the index episode), number of chronic physical illnesses (which required regular outpatient follow ups by other specialties), MMSE scores, referral to other psychiatric disciplines upon discharge (including community psychiatric nurses, clinical psychologists, social workers or day hospital), history of suicidal behaviors (including suicide attempts and self-harm behaviors in lifetime), history of violent behaviors (in lifetime) and number of previous psychiatric admissions.

## Data analysis

### Null hypothesis

The null hypothesis was that none of the identified factors are associated with rehospitalization.

### Univariate tests and multiple logistic regression analysis

After descriptive statistical studies, univariate tests were conducted on all independent variables to identify possible significant factors for subsequent analysis. For categorical factors, a Chi-squared test was performed, and Fisher’s exact test was conducted for factors with an expected cell count less than 5. All the continuous factors in this study were not normally distributed as determined by the Shapiro-Wilk test and Mann-Whitney U test was conducted. Significant and marginally significant factors with a p value of <0.100 identified in the univariate analysis were included in the subsequent multiple logistic regression analysis to identify significant factors (with a p value of <0.050) predicting rehospitalization in 2 years after discharge.

### Survival analysis

Survival analysis was further conducted to identify factors predicting earlier rehospitalization. The rehospitalization rates at different time points within the 2 years were calculated. Univariate analysis with simple Cox regression was performed to all variables. Those with a p value of <0.100 were included in the subsequent multiple Cox regression analysis, to determine variables that significantly (with a p value of <0.050) correlated with time to rehospitalization. Kaplan-Meier survival curves were also plotted.

The protocol of this study was approved by the Hospital Authority’s Research Ethics Committee of Hong Kong East Cluster (Reference number: HKEC-2014-049). Data were analyzed through IBM Statistical Product and Service Solutions (SPSS) software version 22.

## Results

Raw data of elderly patients discharged from psychiatric wards within the study period The coding for each variable is explained in the top row. Blank rows correspond to the discharge episodes that were excluded according to the exclusion criteria. Click here for additional data file. Copyright: © 2015 Wong CYT 2015 Data associated with the article are available under the terms of the Creative Commons Zero "No rights reserved" data waiver (CC0 1.0 Public domain dedication).

**Figure 1.**
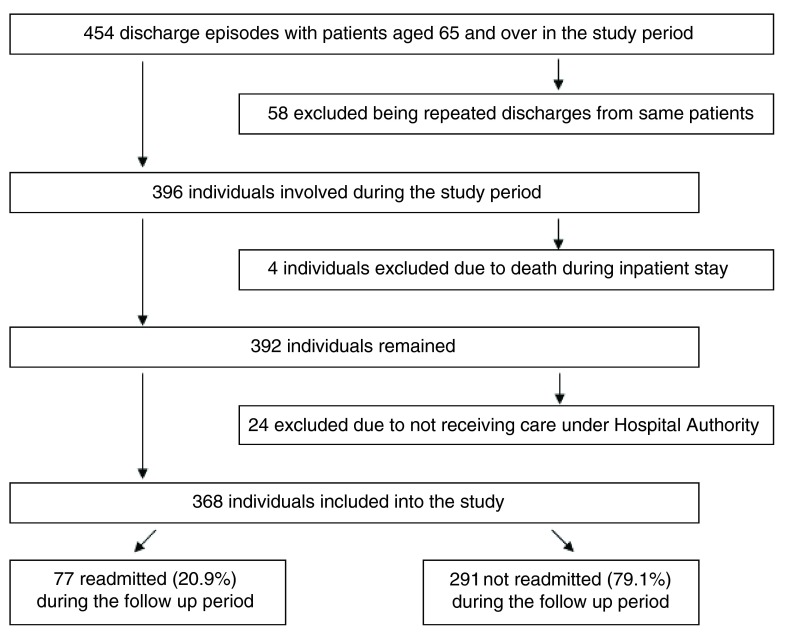
Formation of the study population and separation into the subgroups.

Figure 1 presents the formation of the study population and separation into the subgroups. Of all 454 discharge episodes from psychiatric wards of Pamela Youde Nethersole Eastern Hospital with patients aged 65 and over from 1 March 2010 to 29 February 2012, 368 individuals formed the study population after screening for the exclusion criteria. 77 individuals were readmitted while 291 individuals were not during the 2 year follow up period. The cumulative rehospitalization rate at 2 years was 20.9%.

Concerning the socio-demographic characteristics of the study population, the median age was 76 and male to female ratio was 1 to 1.61. 98.1% of individuals were Chinese. 47% of the study population was married, 39.7% was widowed, 7.9% was divorced or separated and 5.4% was single. For education level, 41.6% was less than primary, 31% was primary, 20.6% was secondary and only 6.8% was tertiary or above. 52.7% of the study population lived at home with family or friends, 33.7% was living in placement, 9% lived alone at home and 4.6% lived with maid at home. Regarding the clinical characteristics of the study population, the median length of inpatient stay for the study population was 27 days. The median for number of chronic physical illnesses was 2 and number of previous admissions was 0. 96.2% of individuals had non-PFU status. For the primary psychiatric diagnosis, 37.2% had cognitive disorders, 32.9% had depressive disorders, 23.1% had psychotic disorders, 4.1% had bipolar disorders and the remaining 2.7% suffered from other mental illnesses. 20.1% of the study population suffered from psychiatric comorbidities. Regarding MMSE scores, 32.3% of the study population scored 11–20, 18.5% scored 21–26, 13.6% scored less than 10, 9% scored over 26, and 26.6% did not have scores documented. 69.8% of individuals had referral to other psychiatric disciplines upon discharge. Lastly, 24.2% had history of suicidal behaviors and 15.8% had history of violence in their lifetime.

Univariate analysis for socio-demographic factors and clinical factors of the study population are shown in Table 1 and Table 2 respectively. Results of subsequent multiple logistic regression are shown in Table 3. Four significant factors were identified with their odds ratios calculated. Referral to other psychiatric disciplines upon discharge (p< 0.001, OR=0.325) was associated with a lower risk of rehospitalization, while the other three factors including history of suicidal behaviors (p< 0.001, OR=4.906), history of violence (p< 0.001, OR=5.443) and number of previous admissions (p< 0.001, OR=1.250) were associated with higher risk of rehospitalization. The overall accuracy of this predictor model was 83.4%.

**Table 1.** Univariate tests for socio-demographic factors.

Socio-demographic Factors	Readmitted N=77		Not readmitted N=291		p
	n	%	n	%	
Gender					
- Male	25	32.5	116	39.9	0.235 ^c^
- Female	52	67.5	175	60.1	
Ethnicity					
- Chinese	74	96.1	287	98.6	0.162 ^f^
- Non-Chinese	3	3.9	4	1.4	
Marital status					0.060 ^c^
- Widow	24	31.2	122	41.9	
- Single	7	9.1	13	4.5	
- Married	36	46.7	137	47.1	
- Divorced or separated	10	13.0	19	6.5	
Education level					0.627 ^c^
- Less than primary	28	36.3	125	43.0
- Primary	24	31.2	90	30.9	
- Secondary	18	23.4	58	19.9	
- Tertiary or above	7	9.1	18	6.2	
Type of residence					0.353 ^c^
- Living alone	10	13.0	23	7.9	
- Home with maid	5	6.5	12	4.1	
- Home with family or friends	40	51.9	154	52.9	
- Living in placement	22	28.6	102	35.1	
	Median	IQR	Median	IQR
Age upon discharge (years)	75	70–80.5	77	72–83	0.050 ^m^

^c^Chi-squared test, ^f^Fisher’s exact test, ^m^Mann Whitney U test, IQR interquartile range

**Table 2.** Univariate tests for clinical factors.

Clinical factors	Readmitted N=77		Not readmitted N=291		p
	n	%	n	%		
PFU status					1.000 ^f^
- Ordinary	74	96.1	280	96.2	
- Target	3	3.9	11	3.8	
Primary psychiatric diagnosis					0.017 ^f^
- Cognitive disorders	26	33.8	111	38.2	
- Depressive disorders	18	23.4	103	35.4	
- Bipolar disorders	3	3.9	12	4.1	
- Psychotic disorders	29	37.6	56	19.2	
- Others	1	1.3	9	3.1	
Psychiatric comorbidities					0.149 ^c^
- Present	20	26.0	54	18.6	
- Absent	57	74.0	237	81.4	
MMSE scores					0.518 ^f^
- Less than 10	14	18.2	36	12.4	
- 11–20	24	31.1	95	32.6	
- 21–26	13	16.9	55	18.9	
- Over 26	4	5.2	29	10.0	
- Not documented	22	28.6	76	26.1	
Referral to other disciplines					0.001 ^c^
- Yes	42	54.5	215	73.9	
- No	35	45.5	76	26.1	
History of suicidal behaviors					0.002 ^c^
- Yes	29	37.7	60	20.6	
- No	48	62.3	231	79.4	
History of violence					<0.001 ^c^
- Yes	28	36.4	30	10.3	
- No	49	63.6	261	89.7	
	Median	IQR	Median	IQR
Length of inpatient stay (days)	30	17–68.5	26	12–50	0.050 ^m^
Number of physical illnesses	2	1–4	2	1–3	0.775 ^m^
Number of previous admissions	1	0–4	0	0–0	<0.001 ^m^

^c^Chi-squared test, ^f^Fisher’s exact test, ^m^Mann Whitney U test

PFU priority follow-up, MMSE Mini-mental State Examination, IQR interquartile range

**Table 3.** Significant factors identified in multiple logistic regression analysis.

Factors	OR	95% CI	p
Referral to other disciplines	0.325	0.174–0.604	<0.001
History of suicidal behaviors	4.906	2.357–10.212	<0.001
History of violence	5.443	2.575–11.506	<0.001
Number of previous admissions	1.250	1.105–1.414	<0.001

Significant p<0.050, OR odds ratio, CI confidence interval

Odds ratios were adjusted by gender and ethnicity

**Figure 2.**
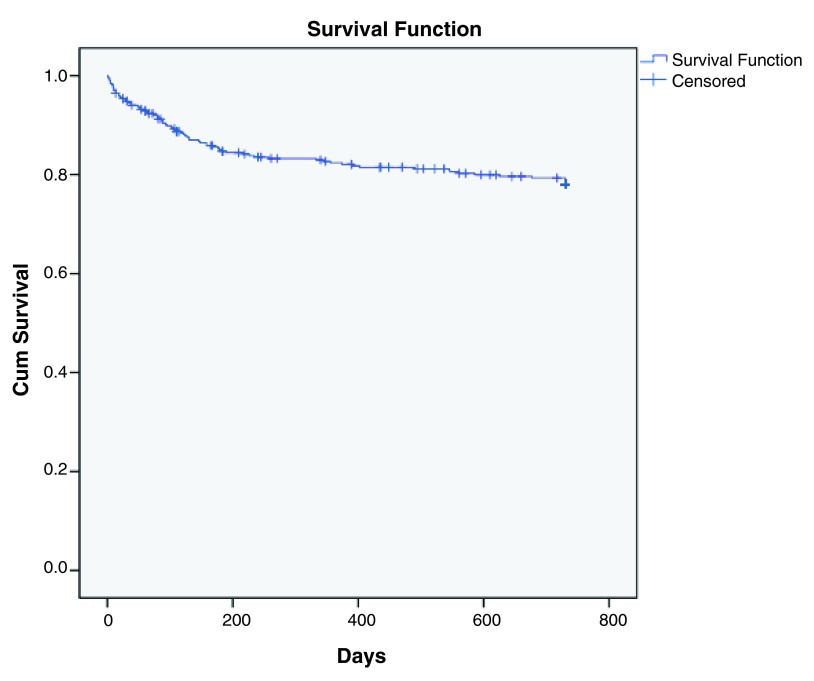
Kaplan-Meier survival curve for graphical representation of the data on rehospitalization.

Survival analysis with Cox regression was then conducted to examine factors that predicted earlier rehospitalization. For individuals who did not readmit but were deceased before completion of the 2 year follow up period, the date of death was taken as the date of censoring. Taking different time points in the Kaplan-Meier survival curve as plotted in Figure 2, the cumulative readmission rate of the study population was 5.2% at 1 month, 9.5% at 3 months, 15.0% at 6 months, 17.1% at 1 year, 18.8% at 1.5 year and lastly 20.9% at 2 years upon completion of the follow up period. Most of the rehospitalization occurred within the first 6 months after discharge where the slope of the curve was steepest.

**Figure 3.**
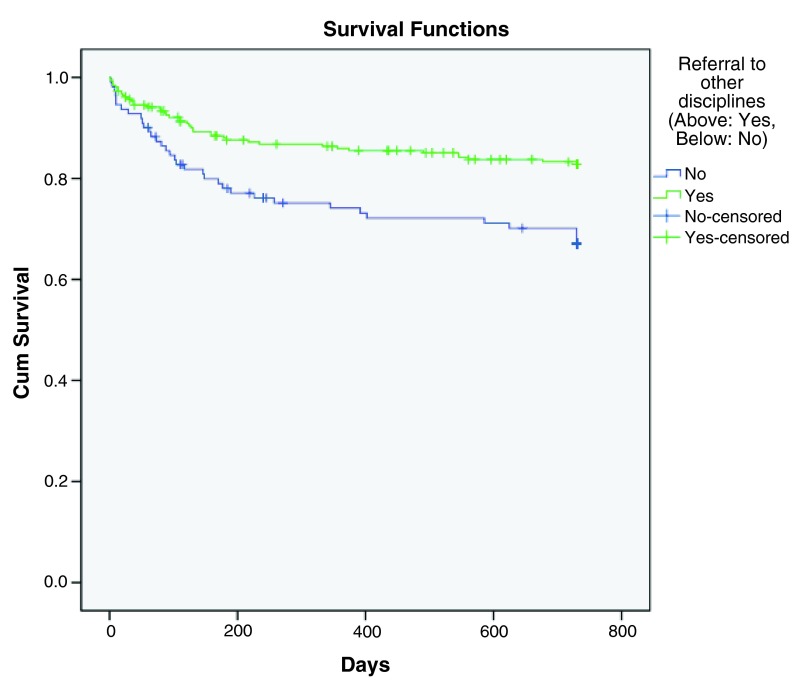
Kaplan-Meier survival curve showing decreased hazard of rehospitalization with referral to other psychiatric disciplines upon discharge.

**Figure 4.**
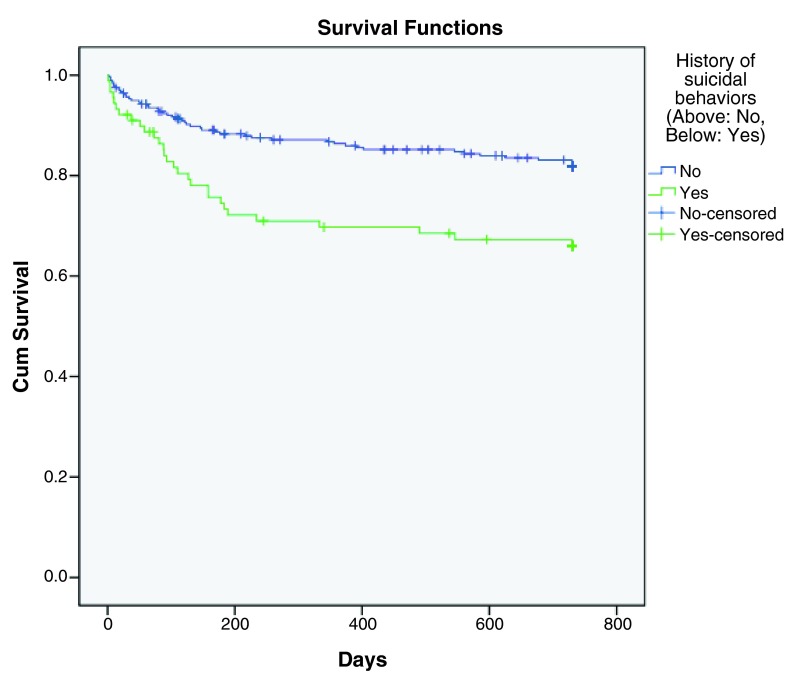
Kaplan-Meier survival curve showing increased hazard of rehospitalization with history of suicidal behaviors.

**Figure 5.**
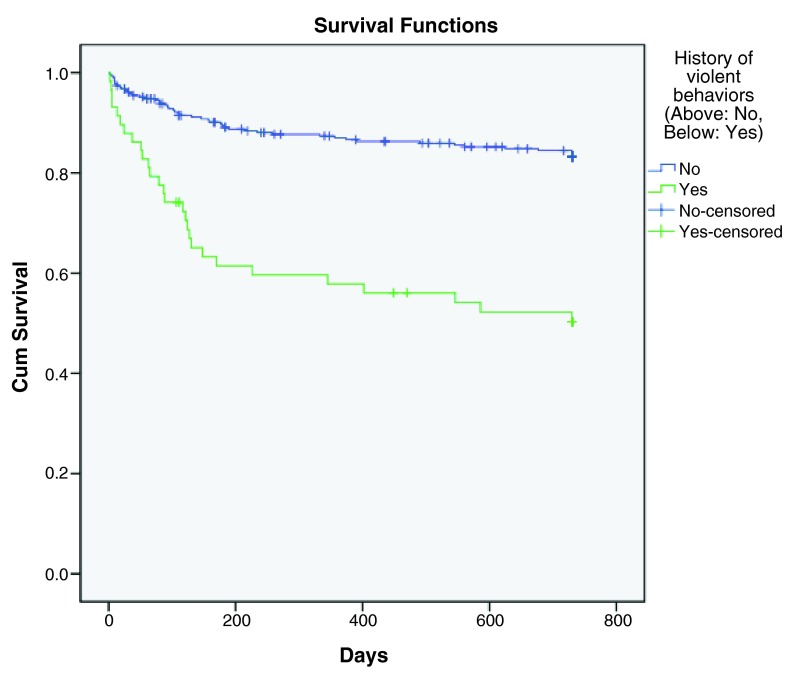
Kaplan-Meier survival curve showing increased hazard of rehospitalization with history of violence.

**Figure 6.**
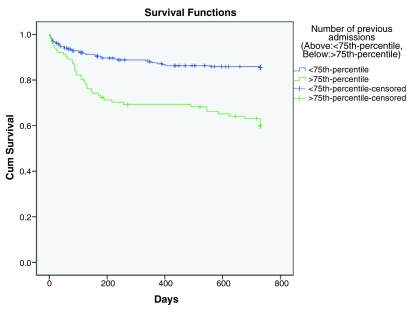
Kaplan-Meier curve survival curve showing increased hazard of rehospitalization with greater number of previous psychiatric admissions.

Univariate analysis by simple Cox regression was performed and the results are shown in Table 4. The significant results of subsequent multiple Cox regression survival analyses are presented in Table 5. The same four significant factors were identified in the multiple Cox regression analysis as that in the previous logistic regression analysis. Hazard ratios were calculated and Kaplan-Meier survival curves were plotted. Referral to other psychiatric disciplines upon discharge (p< 0.001, HR=0.405) correlated to a longer time to rehospitalization ( Figure 3), while history of suicidal behaviors (p< 0.001, HR=3.161), history of violence (p< 0.001, HR=3.935) and number of previous psychiatric admissions (p< 0.001, HR=1.121) were predictive factors for earlier rehospitalization ( Figure 4, Figure 5, Figure 6 respectively).

**Table 4.** Univariate tests with simple Cox regression in survival analysis.

Socio-demographic factors	p
Age	0.103
Gender	0.403
Ethnicity	0.257
Marital status	0.067
Education level	0.722
Type of residence	0.428
Clinical factors	
Priority follow-up status	0.996
Length of inpatient stay	0.470
Primary psychiatric diagnosis	0.042
Presence of psychiatric comorbidities	0.193
Number of chronic physical illnesses	0.662
Mini-mental state examination scores	0.467
Referral to other disciplines	0.001
History of suicidal behaviors	0.001
History of violence	<0.001
Number of previous admissions	<0.001

**Table 5.** Significant factors identified in multiple Cox regression analysis.

Factors	HR	95% CI	p
Referral to other disciplines	0.405	0.248–0.661	<0.001
History of suicidal behaviors	3.161	1.815–5.505	<0.001
History of violence	3.935	2.321–6.670	<0.001
Number of previous admissions	1.121	1.052–1.195	<0.001

Significant p<0.050, HR hazard ratio, CI confidence interval

Hazard ratios were adjusted by age, gender and ethnicity

## Discussion

This study involved Chinese and non-Chinese elderly psychiatric patients in Hong Kong, and by comparison between the two groups, ethnicity was not identified as a significant factor. However, it should be noted that too few cases were non-Chinese and so the statistical comparison between them might not be meaningful. In comparison to other studies involving psychogeriatric patients only, most studies did not find type of residence to be a significant factor in psychiatric readmission, except 1 study ^22^ which reported higher readmission risk in patients with little or no supervision in living arrangements following discharge. However, this study did not find any significant differences in outcome regarding rehospitalization between patients who lived alone, with others, or in placement.

The same four clinical factors were shown to be significantly associated with rehospitalization with a p value of <0.001 in both multiple logistic regression and Cox regression survival analysis, including referral to other psychiatric disciplines upon discharge, history of suicidal behaviors, history of violence and number of previous psychiatric admissions. With adequate aftercare services ^6, 7, 29, 30^ by referral to other disciplines for support upon discharge, patients were more likely to comply with treatment plans and have their problems managed in outpatient settings, which reduced the need for psychiatric rehospitalization. Moreover, a study ^12^ had reported that patients with previous suicidal attempts were more often readmitted, and similar findings had been shown in studies that patients having violence behaviors previously were at higher risk of readmission ^11, 25, 28, 31^. Psychiatric rehospitalization was often required when patients displayed risks of harming self or others. Furthermore, studies had extensively reported that patients with greater number of previous admissions were predisposed to rehospitalization ^7, 8, 15, 20, 25, 32, 33^. These patients might be more often rehospitalized due to suboptimal control on symptoms relating to their mental health problems and inadequate support in the community.

Concerning factors that were more specific to the elderly, 1 reviewed article ^27^ reported that elderly patients without medical comorbidity were more likely rehospitalized, however, number of chronic illnesses was not identified as a significant risk factor. Elderly patients were more likely to be hospitalized due to poor physical conditions. Sometimes psychiatric symptoms had already been treated in general wards, but readmission to general wards were not accounted in this study. Besides, having a cognitive disorder or a lower MMSE score would not affect the risk of rehospitalization. However, it was noted that over one-fourth (26.6%) of the study population did not have their MMSE scores documented, and therefore the results might differ if the MMSE scores from all study individuals were available.

### Limitations

Firstly, this was a retrospective study and the correctness of data depended heavily on the information in CMS. Some variables could have been underestimated or changed during the follow up period. Secondly, the study population involved the psychiatric unit in a regional hospital in Hong Kong only. The findings might not be generalized to other parts of the world.

### Strengths

Data on patients’ characteristics were retrieved through medical records rather than self-reporting from patients and this could minimize recall bias. Besides, this study involved elderly patients only and the findings were more specific towards psychogeriatric patients.

### Implications

These findings were important to the daily practice of psychiatrists and especially the psychogeriatricians, since most of the previous rehospitalization studies in the literature were not specifically targeted to elderly patients. There was very little information available regarding risk factors that predicted psychiatric rehospitalization of elderly patients in Hong Kong prior to this study. In view of the aging population and increasing need for psychiatric services in Hong Kong, this study could help in identifying elderly patients with a high rehospitalization risk for more intensive treatments and better discharge planning based on their risk factors, including history of suicidal behaviors, history of violent behaviors and greater number of previous psychiatric admissions. Factors including cognitive disorders, lower MMSE scores, comorbid chronic physical illnesses, marital status, type of residence, age, gender, ethnicity, PFU status, education level, length of inpatient stay, and presence of psychiatric comorbidities did not affect the risk of rehospitalization in the elderly psychiatric patients. On the other hand, referral to other psychiatric disciplines upon discharge was highly encouraged to prevent rehospitalization.

### Future research

A larger prospective study should be carried out to determine risk factors for psychiatric rehospitalization in elderly patients. Future research could also investigate daily functioning and quality of life of elderly patients for a better understanding on their condition after discharge from psychiatric hospitals.

## Conclusion

This retrospective cohort study has provided important information regarding psychiatric rehospitalization of elderly patients. Rehospitalization was mainly affected by clinical characteristics and occurred mostly within the first 6 months after discharge. Among the significant factors for rehospitalization, history of suicidal behaviors, history of violent behaviors and greater number of previous psychiatric admissions were associated with a higher rehospitalization risk. Referral to other psychiatric disciplines upon discharge predicted a better outcome and was highly recommended for elderly patients.

## Data availability

The data referenced by this article are under copyright with the following copyright statement: Copyright: © 2015 Wong CYT

Data associated with the article are available under the terms of the Creative Commons Zero "No rights reserved" data waiver (CC0 1.0 Public domain dedication). 

*F1000Research*: Dataset 1. Raw data of elderly patients discharged from psychiatric wards within the study period, 10.5256/f1000research.7135.d103399 ^36^

## Appendix

Since 1982, mental patients in Hong Kong were categorized into non-PFU (ordinary), PFU (target) and PFU (subtarget) groups depending on their risk level. Patients in PFU (subtarget) group were considered to have the highest violence risk.
