# Supplementary material for: Predictors of psychiatric rehospitalization among elderly patients
Source: F1000Res. 2015 Sep 30;4:926. [Version 1] doi: 10.12688/f1000research.7135.1 (PMC4732553; doi:10.12688/f1000research.7135.1)
Supplement: Peer review history

# Peer review: Referee response for version 1

**DOI:** 10.5256/f1000research.7685.r11435

**Published:** 2016-05-10

**Authors:** Jeffrey Braithwaite

**Affiliations:** 1 Centre for Healthcare Resilience and Implementation Science, Australian Institute of Health Innovation, Macquarie University, Sydney, NSW, Australia

This was a useful paper and an informative read. The title is clear, although it might be indicated where the study was located (Hong Kong). I personally prefer structured abstracts.

The methods and data analysis are well described. Sensible limitations, including that the study was restricted to one psychiatric unit, are provided. The author makes the valid point that few studies of this type are about elderly patients, and this study fills a gap in our knowledge, although it is confirmatory of much that we already know in the broader domain of psychiatric rehospitalisaton.

I have read this submission. I believe that I have an appropriate level of expertise to confirm that it is of an acceptable scientific standard.

# Peer review: Referee response for version 1

**DOI:** 10.5256/f1000research.7685.r12079

**Published:** 2016-01-25

**Authors:** W Sue Griffin

**Affiliations:** 1 Department of Geriatrics, University of Arkansas for Medical Sciences, Little Rock, AR, USA

In this article, Wong and his colleagues examined several factors that might predict re-hospitalization rates among elderly patients to a psychiatric unit. The study is a retrospective analysis of data from patients mostly at the Pamela Youde Nethersole Eastern Hospital in Hong Kong – possible predictive factors obtained “in part” from previous studies in the same domain were sometimes included. The reported main factors related to re-admission included a history of multiple admissions to hospital, and suicidal and or violent behaviors. It isn’t surprising to note that patients with a history of suicidal or violent behaviors were at greater risk for re-admission as they require “acute” intervention (s). This is important as most re-admissions occurred in the first 6 months after discharge, raising concerns for the need for close outpatient follow up to ensure patient adherence to treatment plans. Importantly, and perhaps related to this, referral of patients to appropriate psychiatric disciplines was associated with fewer re-admissions.

It was surprising that no significant differences were noted in the study, regarding outcomes between patients who lived alone or with others. This, as well as future studies to include patients with chronic psychiatric diseases may allow discoveries regarding relationships between outcomes as they may be affected by inclusion of psychiatric symptoms in patient histories when a patient is admitted to hospital in non-psychiatric wards.

In summary, information reported in this study adds to our limited knowledge regarding the principle reasons for re-admission of elderly patients to hospital and, thus, may lead to improvements in patient care. Moreover, based on outcomes reported in this study, hospitals may benefit by relief from the usual challenges of inpatient bed availability and reimbursement from third party payers. More generally, health care costs might be reduced and family and other caregiver burdens lessened.

I have read this submission. I believe that I have an appropriate level of expertise to confirm that it is of an acceptable scientific standard.

# Response

**Published:** 2016-01-25

**Authors:** Chun Yin Terry Wong

**Affiliations:** Department of Psychiatry, Pamela Youde Nethersole Eastern Hospital, Hong Kong

Thank you so much for your review and your comments.

# Peer review: Referee response for version 1

**DOI:** 10.5256/f1000research.7685.r10954

**Published:** 2015-12-17

**Authors:** Xiao Shifu, Wei Li

**Affiliations:** 1 Alzheimer’s Diagnosis and Treatment Centre, Shanghai Jiaotong University School of Medicine, Shanghai, 200030, China; 2 Alzheimer’s Disease and Related Disorders Center & Shanghai Mental Health Center, Shanghai Jiaotong University School of Medicine, Shanghai, China

We are very interested in this article, for it might be helpful in some clinical practice. However, we need the authors to answer some questions:

  1.  How do you select the possible factors that might be related with re-admission? Why should these preclude the factors of psychiatric drugs;

  2.  We suggest that the measurement data should be represented by mean and its standard deviation.

We have read this submission. We believe that we have an appropriate level of expertise to confirm that it is of an acceptable scientific standard.

# Response

**Published:** 2015-12-17

**Authors:** Chun Yin Terry Wong

**Affiliations:** Department of Psychiatry, Pamela Youde Nethersole Eastern Hospital, Hong Kong

Thanks a lot for your comments. In response to your questions:

1. The possible factors included in the study were the most common predictors of psychiatric rehospitalizations that were identified from the previous studies in the literature. This study investigated if these factors could also affect the risk of rehospitalization in the Chinese elderly psychiatric patients. Since the use of different types of psychiatric drugs had not been shown to be associated with rehospitalization risk in the studies reviewed, this factor was not included as an independent variable.
2. All the continuous variables in the study were not normally distributed. Therefore median and IQR were chosen to represent these data since they would be less affected by the extreme values.
